# Ethyl 6-(6-meth­oxy-2-naphth­yl)-2-oxo-4-(2-thien­yl)cyclo­hex-3-ene-1-carboxyl­ate

**DOI:** 10.1107/S1600536809021308

**Published:** 2009-06-10

**Authors:** Hongqi Li, Anil N. Mayekar, B. Narayana, H. S. Yathirajan, W. T. A. Harrison

**Affiliations:** a Key Laboratory of the Science & Technology of Eco-Textiles, Ministry of Education, College of Chemistry, Chemical Engineering & Biotechnology, Donghua University, Shanghai 201620, People’s Republic of China; b Department of Studies in Chemistry, University of Mysore, Manasagangotri, Mysore 570 006, India; c Department of Studies in Chemistry, Mangalore University, Mangalagangotri 574 199, India; d Department of Chemistry, University of Aberdeen, Aberdeen AB24 3UE, Scotland

## Abstract

The title compound, C_24_H_22_O_4_S, was prepared by reaction between (2*E*)-3-(6-meth­oxy-2-naphth­yl)-1-(2-thien­yl)prop-2-en-1-one and ethyl acetoacetate. In the crystal, the cyclo­hexenone ring shows a distorted half-chair conformation. The length of the double bond in the cyclohexenone ring [1.343 (4) Å] is normal.

## Related literature

For related structures, see: Fischer *et al.* (2007**a* ▶,b* ▶; 2008**a* ▶,b* ▶). For the use of cyclo­hexenones in organic synthesis, see: Padmavathi *et al.* (1999 ▶, 2001 ▶). For pharmaceutical applications of cyclo­hexenone derivatives, see: Hoye & Tennakoon (2000 ▶); Hiromichi *et al.* (2002 ▶).
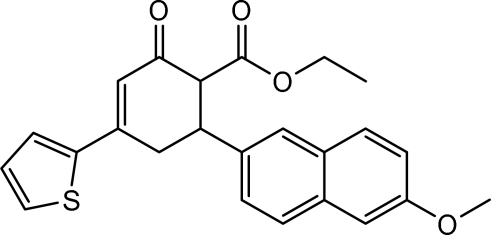


## Experimental

### 

#### Crystal data

C_24_H_22_O_4_S
*M* *_r_* = 406.48
Monoclinic, 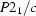

*a* = 18.2501 (4) Å
*b* = 11.7176 (2) Å
*c* = 9.6846 (2) Å
β = 93.048 (1)°
*V* = 2068.10 (7) Å^3^
*Z* = 4
Mo *K*α radiation
μ = 0.18 mm^−1^
*T* = 296 K
0.45 × 0.29 × 0.16 mm

#### Data collection

Bruker SMART CCD area-detector diffractometer
Absorption correction: multi-scan (*SADABS*; Sheldrick, 2004 ▶) *T*_min_ = 0.922, *T*_max_ = 0.972
17446 measured reflections
4035 independent reflections
2880 reflections with *I* > 2σ(*I*)
*R*_int_ = 0.028

#### Refinement

*R*[*F* ^2^ > 2σ(*F* ^2^)] = 0.063
*wR*(*F* ^2^) = 0.202
*S* = 1.02
4035 reflections
282 parameters
H-atom parameters constrained
Δρ_max_ = 0.26 e Å^−3^
Δρ_min_ = −0.38 e Å^−3^

### 

Data collection: *SMART* (Bruker, 2001 ▶); cell refinement: *SAINT* (Bruker, 2001 ▶); data reduction: *SAINT*; program(s) used to solve structure: *SHELXS97* (Sheldrick, 2008 ▶); program(s) used to refine structure: *SHELXL97* (Sheldrick, 2008 ▶); molecular graphics: *SHELXTL* (Sheldrick, 2008 ▶); software used to prepare material for publication: *SHELXTL*.

## Supplementary Material

Crystal structure: contains datablocks global, I. DOI: 10.1107/S1600536809021308/sj2629sup1.cif

Structure factors: contains datablocks I. DOI: 10.1107/S1600536809021308/sj2629Isup2.hkl

Additional supplementary materials:  crystallographic information; 3D view; checkCIF report

## Figures and Tables

**Fig. 1.**
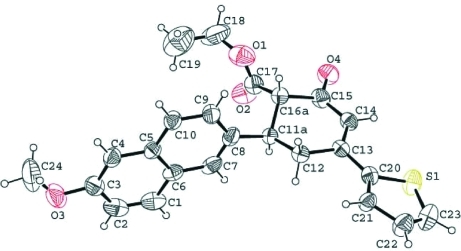
A view of the crystal structure of the title compound.

## supplementary crystallographic information

### Comment

Cyclohexenones are efficient synthons in building spiro compounds (Padmavathi *et al.*, 2001) or intermediates in the synthesis of benzisoxazole or carbazole derivatives (Padmavathi *et al.*, 1999). Cyclohexenone derivatives are well known lead compounds for the treatment of inflammation and autoimmune diseases (Hoye & Tennakoon, 2000; Hiromichi *et al.*, 2002).

The crystal structures of a series of ethyl 6-substituted 2-oxocyclohex- 3-ene-1-carboxylates have been reported (Fischer *et al.*, 2007; 2007*a*,*b*; 2008*a**b*). In view of the importance of these derivatives and continuing our efforts in this field, the title compound, ethyl 6-(6- methoxynaphthalen-2-yl)-2-oxo-4-(2-thienyl)cyclohexa-3-ene-1- carboxylate, was synthesized and its crystal structure is reported in this paper.

### Experimental

(2E)-3-(6-methoxy-2-naphthyl)-1-(2-thienyl)prop-2-en-1-one (1.51 g, 5 mmol) and ethyl acetoacetate (0.65 g, 5 mmol) were refluxed for 6 hr in 10–15 ml of ethanol in the presence of 0.8 ml 10% NaOH. The reaction mixture was cooled to room temperature and the resulting product was filtered and recrystallized from acetonitrile (m.p.: 415–418 K). Analysis % found (calculated): C, 71.19 (71.27); H, 4.94 (4.98); S, 7.89 (7.93).

### Refinement

All H atoms were placed in idealized locations (C—H = 0.93–0.98 Å) and refined as riding with *U*_iso_(H) = 1.2*U*_eq_(C) or 1.5*U*_eq_(methyl C). The methyl groups were allowed to rotate, but not to tip, to best fit the electron density. The large difference between min and max *U*_iso_ values for the H atoms is a result of  unresolved disorder in the ethyl side chain.

### Crystal data

**Table:**

C_24_H_22_O_4_S	*F*(000) = 856
*M**_r_* = 406.48	*D*_x_ = 1.305 Mg m^−^^3^
Monoclinic, *P*2_1_/*c*	Melting point = 415–418 K
Hall symbol: -P 2ybc	Mo *K*α radiation, λ = 0.71073 Å
*a* = 18.2501 (4) Å	Cell parameters from 8575 reflections
*b* = 11.7176 (2) Å	θ = 2.3–28.1°
*c* = 9.6846 (2) Å	µ = 0.18 mm^−^^1^
β = 93.048 (1)°	*T* = 296 K
*V* = 2068.10 (7) Å^3^	Block, colourless
*Z* = 4	0.45 × 0.29 × 0.16 mm

### Data collection

**Table:**

Bruker SMART CCD area-detector diffractometer	4035 independent reflections
Radiation source: fine-focus sealed tube	2880 reflections with *I* > 2σ(*I*)
graphite	*R*_int_ = 0.028
φ and ω scans	θ_max_ = 26.0°, θ_min_ = 2.2°
Absorption correction: multi-scan (*SADABS*; Sheldrick, 2004)	*h* = −22→22
*T*_min_ = 0.922, *T*_max_ = 0.972	*k* = −11→14
17446 measured reflections	*l* = −11→10

### Refinement

**Table:**

Refinement on *F*^2^	Primary atom site location: structure-invariant direct methods
Least-squares matrix: full	Secondary atom site location: difference Fourier map
*R*[*F*^2^ > 2σ(*F*^2^)] = 0.063	Hydrogen site location: inferred from neighbouring sites
*wR*(*F*^2^) = 0.202	H-atom parameters constrained
*S* = 1.02	*w* = 1/[σ^2^(*F*_o_^2^) + (0.095*P*)^2^ + 0.9907*P*] where *P* = (*F*_o_^2^ + 2*F*_c_^2^)/3
4035 reflections	(Δ/σ)_max_ = 0.001
282 parameters	Δρ_max_ = 0.26 e Å^−^^3^
0 restraints	Δρ_min_ = −0.37 e Å^−^^3^

### Special details

**Table:**

Geometry. All e.s.d.'s (except the e.s.d. in the dihedral angle between two l.s. planes) are estimated using the full covariance matrix. The cell e.s.d.'s are taken into account individually in the estimation of e.s.d.'s in distances, angles and torsion angles; correlations between e.s.d.'s in cell parameters are only used when they are defined by crystal symmetry. An approximate (isotropic) treatment of cell e.s.d.'s is used for estimating e.s.d.'s involving l.s. planes.
Refinement. Refinement of *F*^2^ against ALL reflections. The weighted *R*-factor *wR* and goodness of fit *S* are based on *F*^2^, conventional *R*-factors *R* are based on *F*, with *F* set to zero for negative *F*^2^. The threshold expression of *F*^2^ > σ(*F*^2^) is used only for calculating *R*-factors(gt) *etc*. and is not relevant to the choice of reflections for refinement. *R*-factors based on *F*^2^ are statistically about twice as large as those based on *F*, and *R*- factors based on ALL data will be even larger.

### Fractional atomic coordinates and isotropic or equivalent isotropic displacement parameters (Å^2^)

**Table:**

	*x*	*y*	*z*	*U*_iso_*/*U*_eq_	Occ. (<1)
C1	0.1379 (2)	0.5193 (3)	0.4085 (3)	0.0917 (10)	
H1A	0.1524	0.4568	0.4625	0.110*	
C2	0.0777 (2)	0.5795 (4)	0.4405 (4)	0.0955 (12)	
H2A	0.0516	0.5577	0.5160	0.115*	
C3	0.05423 (16)	0.6739 (3)	0.3619 (4)	0.0845 (9)	
C4	0.09205 (15)	0.7057 (3)	0.2525 (3)	0.0733 (8)	
H4A	0.0762	0.7682	0.1997	0.088*	
C5	0.15546 (13)	0.6457 (2)	0.2168 (3)	0.0593 (6)	
C6	0.17879 (16)	0.5499 (2)	0.2952 (3)	0.0661 (7)	
C7	0.24126 (19)	0.4896 (3)	0.2584 (3)	0.0810 (9)	
H7A	0.2567	0.4267	0.3108	0.097*	
C8	0.28020 (16)	0.5208 (3)	0.1474 (3)	0.0787 (9)	
C9	0.25672 (16)	0.6164 (3)	0.0705 (3)	0.0768 (8)	
H9A	0.2829	0.6392	−0.0045	0.092*	
C10	0.19634 (15)	0.6766 (3)	0.1033 (3)	0.0694 (7)	
H10A	0.1818	0.7393	0.0497	0.083*	
C11A	0.3387 (4)	0.4303 (7)	0.1361 (8)	0.0567 (17)	0.489 (11)
H11A	0.3274	0.3649	0.1944	0.068*	0.489 (11)
C11B	0.3537 (3)	0.4735 (6)	0.0866 (7)	0.0498 (15)	0.511 (11)
H11B	0.3642	0.5140	0.0013	0.060*	0.511 (11)
C12	0.41517 (14)	0.4837 (2)	0.1877 (3)	0.0587 (6)	
H12A	0.4212	0.5568	0.1426	0.070*	
H12B	0.4149	0.4974	0.2864	0.070*	
C13	0.47940 (14)	0.4085 (2)	0.1587 (3)	0.0563 (6)	
C14	0.47374 (16)	0.3283 (2)	0.0596 (3)	0.0654 (7)	
H14A	0.5156	0.2875	0.0396	0.078*	
C15	0.40620 (18)	0.3025 (3)	−0.0168 (3)	0.0779 (8)	
C16A	0.3403 (3)	0.3921 (6)	−0.0137 (8)	0.0659 (19)	0.511 (11)
H16A	0.3472	0.4565	−0.0763	0.079*	0.511 (11)
C16B	0.3388 (3)	0.3456 (5)	0.0561 (7)	0.0501 (17)	0.489 (11)
H16B	0.3323	0.3031	0.1418	0.060*	0.489 (11)
C17	0.27068 (18)	0.3302 (3)	−0.0483 (4)	0.0771 (8)	
C18	0.1848 (5)	0.3678 (7)	−0.2222 (6)	0.190 (3)	
H18A	0.1700	0.2910	−0.1987	0.228*	
H18B	0.1906	0.3710	−0.3211	0.228*	
C19	0.1297 (5)	0.4479 (7)	−0.1848 (8)	0.212 (3)	
H19A	0.0839	0.4294	−0.2329	0.317*	
H19B	0.1442	0.5236	−0.2095	0.317*	
H19C	0.1240	0.4443	−0.0869	0.317*	
C20	0.54589 (14)	0.4261 (2)	0.2453 (3)	0.0628 (7)	
C21	0.56318 (14)	0.5200 (2)	0.3318 (3)	0.0689 (7)	
H21A	0.5331	0.5831	0.3424	0.083*	
C22	0.63359 (19)	0.5044 (3)	0.4008 (4)	0.0956 (10)	
H22A	0.6556	0.5588	0.4595	0.115*	
C23	0.66455 (19)	0.4056 (3)	0.3736 (5)	0.1036 (12)	
H23A	0.7098	0.3828	0.4128	0.124*	
C24	−0.0321 (3)	0.8260 (6)	0.3341 (8)	0.169 (3)	
H24A	−0.0767	0.8524	0.3713	0.254*	
H24B	−0.0408	0.8082	0.2379	0.254*	
H24C	0.0047	0.8844	0.3443	0.254*	
S1	0.61390 (5)	0.32512 (7)	0.26068 (12)	0.0945 (4)	
O1	0.2523 (2)	0.3953 (3)	−0.1501 (3)	0.1250 (10)	
O2	0.23644 (17)	0.2511 (2)	−0.0137 (3)	0.1130 (9)	
O3	−0.00726 (14)	0.7259 (3)	0.4069 (3)	0.1238 (11)	
O4	0.40154 (14)	0.2313 (2)	−0.1083 (2)	0.0996 (8)	

### Atomic displacement parameters (Å^2^)

**Table:**

	*U*^11^	*U*^22^	*U*^33^	*U*^12^	*U*^13^	*U*^23^
C1	0.127 (3)	0.076 (2)	0.070 (2)	−0.028 (2)	−0.0062 (19)	0.0070 (16)
C2	0.106 (3)	0.110 (3)	0.071 (2)	−0.048 (2)	0.0134 (18)	−0.006 (2)
C3	0.0610 (16)	0.109 (3)	0.084 (2)	−0.0190 (16)	0.0062 (14)	−0.015 (2)
C4	0.0597 (15)	0.080 (2)	0.0794 (18)	0.0007 (13)	−0.0029 (13)	−0.0023 (15)
C5	0.0576 (13)	0.0555 (15)	0.0637 (14)	−0.0041 (11)	−0.0067 (11)	−0.0018 (12)
C6	0.0832 (17)	0.0517 (15)	0.0614 (15)	−0.0115 (13)	−0.0138 (13)	−0.0029 (12)
C7	0.104 (2)	0.0579 (17)	0.077 (2)	0.0198 (16)	−0.0339 (17)	−0.0135 (15)
C8	0.0733 (17)	0.079 (2)	0.080 (2)	0.0214 (15)	−0.0240 (15)	−0.0337 (17)
C9	0.0673 (16)	0.085 (2)	0.0781 (18)	0.0060 (15)	0.0041 (14)	−0.0083 (16)
C10	0.0680 (16)	0.0662 (18)	0.0738 (17)	0.0069 (13)	0.0020 (13)	0.0082 (14)
C11A	0.067 (3)	0.047 (4)	0.056 (4)	−0.003 (3)	0.002 (3)	0.006 (3)
C11B	0.062 (3)	0.037 (3)	0.050 (3)	0.001 (2)	0.003 (2)	0.005 (2)
C12	0.0691 (14)	0.0452 (13)	0.0620 (14)	0.0002 (11)	0.0051 (11)	−0.0005 (11)
C13	0.0683 (14)	0.0429 (13)	0.0588 (14)	0.0007 (10)	0.0134 (11)	0.0065 (11)
C14	0.0757 (16)	0.0535 (15)	0.0682 (16)	0.0077 (12)	0.0151 (13)	−0.0001 (13)
C15	0.089 (2)	0.0624 (17)	0.0824 (19)	0.0045 (15)	0.0099 (15)	−0.0215 (16)
C16A	0.085 (4)	0.052 (4)	0.061 (4)	−0.002 (3)	0.003 (3)	0.002 (3)
C16B	0.068 (3)	0.037 (3)	0.045 (3)	−0.002 (2)	0.001 (2)	0.001 (3)
C17	0.0830 (19)	0.0625 (19)	0.086 (2)	−0.0068 (15)	0.0024 (16)	−0.0232 (17)
C18	0.266 (8)	0.195 (6)	0.103 (4)	0.108 (6)	−0.067 (5)	−0.030 (4)
C19	0.222 (8)	0.195 (7)	0.209 (7)	0.017 (7)	−0.066 (6)	−0.024 (6)
C20	0.0628 (14)	0.0547 (15)	0.0719 (16)	0.0001 (11)	0.0139 (12)	0.0094 (13)
C21	0.0631 (14)	0.0668 (17)	0.0753 (17)	0.0019 (12)	−0.0092 (12)	−0.0095 (14)
C22	0.084 (2)	0.090 (2)	0.110 (3)	−0.0001 (19)	−0.0164 (19)	−0.011 (2)
C23	0.0742 (19)	0.091 (3)	0.144 (3)	−0.0013 (18)	−0.015 (2)	0.014 (2)
C24	0.101 (3)	0.190 (6)	0.220 (6)	0.053 (4)	0.038 (4)	−0.022 (5)
S1	0.0799 (5)	0.0638 (5)	0.1391 (9)	0.0073 (4)	−0.0014 (5)	0.0047 (5)
O1	0.171 (3)	0.105 (2)	0.100 (2)	0.004 (2)	0.022 (2)	0.0166 (17)
O2	0.140 (2)	0.0862 (18)	0.1104 (19)	−0.0330 (17)	−0.0168 (17)	0.0061 (15)
O3	0.0724 (15)	0.181 (3)	0.120 (2)	−0.0047 (18)	0.0278 (15)	−0.032 (2)
O4	0.1168 (18)	0.0868 (16)	0.0945 (16)	0.0164 (13)	−0.0023 (13)	−0.0425 (14)

### Geometric parameters (Å, °)

**Table:**

C1—C2	1.354 (5)	C14—C15	1.436 (4)
C1—C6	1.406 (4)	C14—H14A	0.9300
C1—H1A	0.9300	C15—O4	1.217 (3)
C2—C3	1.396 (5)	C15—C16B	1.536 (6)
C2—H2A	0.9300	C15—C16A	1.599 (7)
C3—C4	1.347 (4)	C16A—C17	1.486 (7)
C3—O3	1.369 (4)	C16A—H16A	0.9800
C4—C5	1.413 (4)	C16B—C17	1.571 (6)
C4—H4A	0.9300	C16B—H16B	0.9800
C5—C10	1.408 (4)	C17—O2	1.176 (4)
C5—C6	1.409 (4)	C17—O1	1.277 (4)
C6—C7	1.403 (4)	C18—O1	1.420 (7)
C7—C8	1.370 (5)	C18—C19	1.437 (9)
C7—H7A	0.9300	C18—H18A	0.9700
C8—C9	1.400 (5)	C18—H18B	0.9700
C8—C11A	1.513 (7)	C19—H19A	0.9600
C8—C11B	1.593 (6)	C19—H19B	0.9600
C9—C10	1.360 (4)	C19—H19C	0.9600
C9—H9A	0.9300	C20—C21	1.409 (4)
C10—H10A	0.9300	C20—S1	1.715 (3)
C11A—C16A	1.520 (8)	C21—C22	1.428 (4)
C11A—C12	1.586 (7)	C21—H21A	0.9300
C11A—H11A	0.9800	C22—C23	1.321 (5)
C11B—C12	1.455 (6)	C22—H22A	0.9300
C11B—C16B	1.549 (7)	C23—S1	1.683 (4)
C11B—H11B	0.9800	C23—H23A	0.9300
C12—C13	1.505 (3)	C24—O3	1.429 (7)
C12—H12A	0.9700	C24—H24A	0.9600
C12—H12B	0.9700	C24—H24B	0.9600
C13—C14	1.343 (4)	C24—H24C	0.9600
C13—C20	1.453 (4)		
			
C2—C1—C6	121.1 (3)	C13—C14—H14A	118.4
C2—C1—H1A	119.5	C15—C14—H14A	118.4
C6—C1—H1A	119.5	O4—C15—C14	123.0 (3)
C1—C2—C3	121.1 (3)	O4—C15—C16B	122.3 (3)
C1—C2—H2A	119.4	C14—C15—C16B	112.3 (3)
C3—C2—H2A	119.4	O4—C15—C16A	116.1 (3)
C4—C3—O3	126.1 (4)	C14—C15—C16A	118.6 (3)
C4—C3—C2	119.5 (3)	C16B—C15—C16A	32.2 (2)
O3—C3—C2	114.4 (3)	C17—C16A—C11A	107.4 (5)
C3—C4—C5	121.0 (3)	C17—C16A—C15	108.1 (4)
C3—C4—H4A	119.5	C11A—C16A—C15	105.3 (5)
C5—C4—H4A	119.5	C17—C16A—H16A	111.9
C10—C5—C6	117.9 (2)	C11A—C16A—H16A	111.9
C10—C5—C4	122.6 (3)	C15—C16A—H16A	111.9
C6—C5—C4	119.5 (3)	C15—C16B—C11B	105.6 (4)
C7—C6—C1	122.8 (3)	C15—C16B—C17	107.0 (4)
C7—C6—C5	119.4 (3)	C11B—C16B—C17	111.0 (4)
C1—C6—C5	117.8 (3)	C15—C16B—H16B	111.0
C8—C7—C6	121.8 (3)	C11B—C16B—H16B	111.0
C8—C7—H7A	119.1	C17—C16B—H16B	111.0
C6—C7—H7A	119.1	O2—C17—O1	124.7 (3)
C7—C8—C9	118.3 (3)	O2—C17—C16A	141.4 (5)
C7—C8—C11A	105.5 (5)	O1—C17—C16A	93.9 (5)
C9—C8—C11A	136.0 (5)	O2—C17—C16B	108.7 (4)
C7—C8—C11B	132.8 (4)	O1—C17—C16B	126.5 (4)
C9—C8—C11B	108.8 (4)	C16A—C17—C16B	32.9 (3)
C11A—C8—C11B	28.1 (2)	O1—C18—C19	109.2 (5)
C10—C9—C8	121.3 (3)	O1—C18—H18A	109.8
C10—C9—H9A	119.3	C19—C18—H18A	109.8
C8—C9—H9A	119.3	O1—C18—H18B	109.8
C9—C10—C5	121.2 (3)	C19—C18—H18B	109.8
C9—C10—H10A	119.4	H18A—C18—H18B	108.3
C5—C10—H10A	119.4	C18—C19—H19A	109.5
C8—C11A—C16A	108.9 (5)	C18—C19—H19B	109.5
C8—C11A—C12	108.2 (5)	H19A—C19—H19B	109.5
C16A—C11A—C12	110.9 (5)	C18—C19—H19C	109.5
C8—C11A—H11A	109.6	H19A—C19—H19C	109.5
C16A—C11A—H11A	109.6	H19B—C19—H19C	109.5
C12—C11A—H11A	109.6	C21—C20—C13	127.4 (2)
C12—C11B—C16B	109.2 (4)	C21—C20—S1	110.4 (2)
C12—C11B—C8	110.9 (4)	C13—C20—S1	122.1 (2)
C16B—C11B—C8	105.4 (5)	C20—C21—C22	110.3 (3)
C12—C11B—H11B	110.4	C20—C21—H21A	124.9
C16B—C11B—H11B	110.4	C22—C21—H21A	124.9
C8—C11B—H11B	110.4	C23—C22—C21	113.7 (3)
C11B—C12—C13	114.0 (3)	C23—C22—H22A	123.2
C11B—C12—C11A	28.5 (2)	C21—C22—H22A	123.2
C13—C12—C11A	113.0 (3)	C22—C23—S1	113.3 (3)
C11B—C12—H12A	82.6	C22—C23—H23A	123.4
C13—C12—H12A	109.0	S1—C23—H23A	123.4
C11A—C12—H12A	109.0	O3—C24—H24A	109.5
C11B—C12—H12B	129.3	O3—C24—H24B	109.5
C13—C12—H12B	109.0	H24A—C24—H24B	109.5
C11A—C12—H12B	109.0	O3—C24—H24C	109.5
H12A—C12—H12B	107.8	H24A—C24—H24C	109.5
C14—C13—C20	122.8 (2)	H24B—C24—H24C	109.5
C14—C13—C12	120.8 (2)	C23—S1—C20	92.28 (17)
C20—C13—C12	116.4 (2)	C17—O1—C18	115.4 (4)
C13—C14—C15	123.2 (3)	C3—O3—C24	116.9 (3)
			
C6—C1—C2—C3	0.1 (5)	C13—C14—C15—C16B	−18.7 (5)
C1—C2—C3—C4	−0.1 (5)	C13—C14—C15—C16A	16.4 (6)
C1—C2—C3—O3	−180.0 (3)	C8—C11A—C16A—C17	−68.1 (6)
O3—C3—C4—C5	179.3 (3)	C12—C11A—C16A—C17	172.9 (6)
C2—C3—C4—C5	−0.5 (5)	C8—C11A—C16A—C15	176.9 (6)
C3—C4—C5—C10	−180.0 (3)	C12—C11A—C16A—C15	57.9 (6)
C3—C4—C5—C6	1.1 (4)	O4—C15—C16A—C17	39.2 (7)
C2—C1—C6—C7	−179.8 (3)	C14—C15—C16A—C17	−157.4 (4)
C2—C1—C6—C5	0.5 (4)	C16B—C15—C16A—C17	−70.5 (7)
C10—C5—C6—C7	0.3 (4)	O4—C15—C16A—C11A	153.7 (4)
C4—C5—C6—C7	179.2 (2)	C14—C15—C16A—C11A	−42.9 (6)
C10—C5—C6—C1	179.9 (3)	C16B—C15—C16A—C11A	44.1 (5)
C4—C5—C6—C1	−1.1 (4)	O4—C15—C16B—C11B	−144.5 (4)
C1—C6—C7—C8	179.9 (3)	C14—C15—C16B—C11B	52.6 (5)
C5—C6—C7—C8	−0.4 (4)	C16A—C15—C16B—C11B	−55.9 (5)
C6—C7—C8—C9	0.7 (4)	O4—C15—C16B—C17	−26.2 (7)
C6—C7—C8—C11A	−175.6 (3)	C14—C15—C16B—C17	170.9 (3)
C6—C7—C8—C11B	176.6 (3)	C16A—C15—C16B—C17	62.4 (6)
C7—C8—C9—C10	−0.7 (4)	C12—C11B—C16B—C15	−66.7 (5)
C11A—C8—C9—C10	174.1 (4)	C8—C11B—C16B—C15	174.1 (5)
C11B—C8—C9—C10	−177.6 (3)	C12—C11B—C16B—C17	177.7 (5)
C8—C9—C10—C5	0.6 (5)	C8—C11B—C16B—C17	58.5 (5)
C6—C5—C10—C9	−0.4 (4)	C11A—C16A—C17—O2	−58.3 (7)
C4—C5—C10—C9	−179.3 (3)	C15—C16A—C17—O2	54.8 (8)
C7—C8—C11A—C16A	132.9 (5)	C11A—C16A—C17—O1	123.3 (5)
C9—C8—C11A—C16A	−42.3 (8)	C15—C16A—C17—O1	−123.5 (5)
C11B—C8—C11A—C16A	−59.2 (7)	C11A—C16A—C17—C16B	−48.7 (5)
C7—C8—C11A—C12	−106.5 (5)	C15—C16A—C17—C16B	64.4 (6)
C9—C8—C11A—C12	78.3 (6)	C15—C16B—C17—O2	104.7 (5)
C11B—C8—C11A—C12	61.4 (7)	C11B—C16B—C17—O2	−140.6 (4)
C7—C8—C11B—C12	−60.7 (7)	C15—C16B—C17—O1	−78.9 (5)
C9—C8—C11B—C12	115.5 (5)	C11B—C16B—C17—O1	35.9 (6)
C11A—C8—C11B—C12	−76.8 (8)	C15—C16B—C17—C16A	−69.0 (6)
C7—C8—C11B—C16B	57.3 (5)	C11B—C16B—C17—C16A	45.8 (5)
C9—C8—C11B—C16B	−126.5 (4)	C14—C13—C20—C21	166.3 (3)
C11A—C8—C11B—C16B	41.2 (6)	C12—C13—C20—C21	−15.2 (4)
C16B—C11B—C12—C13	45.4 (6)	C14—C13—C20—S1	−17.2 (3)
C8—C11B—C12—C13	161.1 (4)	C12—C13—C20—S1	161.28 (18)
C16B—C11B—C12—C11A	−49.0 (6)	C13—C20—C21—C22	179.4 (3)
C8—C11B—C12—C11A	66.7 (8)	S1—C20—C21—C22	2.6 (3)
C8—C11A—C12—C11B	−71.9 (8)	C20—C21—C22—C23	−2.8 (5)
C16A—C11A—C12—C11B	47.5 (6)	C21—C22—C23—S1	1.7 (5)
C8—C11A—C12—C13	−170.2 (4)	C22—C23—S1—C20	−0.1 (3)
C16A—C11A—C12—C13	−50.8 (6)	C21—C20—S1—C23	−1.5 (2)
C11B—C12—C13—C14	−9.4 (5)	C13—C20—S1—C23	−178.5 (2)
C11A—C12—C13—C14	21.7 (5)	O2—C17—O1—C18	0.9 (6)
C11B—C12—C13—C20	172.1 (4)	C16A—C17—O1—C18	179.6 (4)
C11A—C12—C13—C20	−156.9 (4)	C16B—C17—O1—C18	−175.0 (4)
C20—C13—C14—C15	173.4 (3)	C19—C18—O1—C17	104.5 (7)
C12—C13—C14—C15	−5.0 (4)	C4—C3—O3—C24	−2.5 (6)
C13—C14—C15—O4	178.6 (3)	C2—C3—O3—C24	177.4 (4)

## References

[bb1] Bruker (2001). *SMART* and *SAINT* Bruker AXS Inc., Madison, Wisconsin, USA.

[bb2] Fischer, A., Swamy, M. T., Narayana, B. & Yathirajan, H. S. (2008*a*). *Acta Cryst.* E**64**, o2152. 10.1107/S1600536808032650 PMC2959655 21581012

[bb3] Fischer, A., Yathirajan, H. S., Ashalatha, B. V., Narayana, B. & Sarojini, B. K. (2007*a*). *Acta Cryst.* E**63**, o254–o255. 10.1107/S1600536808002717 PMC2960782 21201903

[bb4] Fischer, A., Yathirajan, H. S., Ashalatha, B. V., Narayana, B. & Sarojini, B. K. (2007*b*). *Acta Cryst.* E**63**, o3616. 10.1107/S1600536808002717 PMC2960782 21201903

[bb5] Fischer, A., Yathirajan, H. S., Ashalatha, B. V., Narayana, B. & Sarojini, B. K. (2008*b*). *Acta Cryst.* E**64**, o560. 10.1107/S1600536808002717 PMC2960782 21201903

[bb6] Hiromichi, F., Naoyuki, K., Yoshinari, S., Yasushi, N. & Yasuyuki, K. (2002). *Tetrahedron Lett.* **43**, 4825–4828.

[bb7] Hoye, T. R. & Tennakoon, M. A. (2000). *Org. Lett.* **2**, 1481–1483. 10.1021/ol0058386 10814478

[bb8] Padmavathi, V., Sharmila, K., Padmaja, A. & Bhaskar Reddy, D. (1999). *Heterocycl. Commun.* **5**, 451–456.

[bb9] Padmavathi, V., Sharmila, K., Somashekara Reddy, A. & Bhaskar Reddy, D. (2001). *Ind. J. Chem. Sect. B*, **40**, 11–14.

[bb10] Sheldrick, G. M. (2004). *SADABS* University of Göttingen, Germany.

[bb11] Sheldrick, G. M. (2008). *Acta Cryst.* A**64**, 112–122. 10.1107/S0108767307043930 18156677

